# Plasma sex hormones and risk of conventional and serrated precursors of colorectal cancer in postmenopausal women

**DOI:** 10.1186/s12916-020-01895-1

**Published:** 2021-01-28

**Authors:** Dong Hang, Xiaosheng He, Ane Sørlie Kværner, Andrew T. Chan, Kana Wu, Shuji Ogino, Zhibin Hu, Hongbing Shen, Edward L. Giovannucci, Mingyang Song

**Affiliations:** 1 Department of Epidemiology and Biostatistics, Jiangsu Key Lab of Cancer Biomarkers, Prevention and Treatment, Collaborative Innovation Center for Cancer Personalized Medicine, School of Public Health, Nanjing Medical University, Nanjing, China; 2 Department of Nutrition, Harvard T.H. Chan School of Public Health, 667 Huntington Avenue, Kresge 906A, Boston, MA 02115 USA; 3 Clinical and Translational Epidemiology Unit and Division of Gastroenterology, Massachusetts General Hospital and Harvard Medical School, Boston, MA USA; 4 Department of Colorectal Surgery, The Six Affiliated Hospital, Sun Yat-sen University, Guangzhou, China; 5 Department of Nutrition, Institute of Basic Medical Sciences, University of Oslo, Oslo, Norway; 6 Channing Division of Network Medicine, Department of Medicine, Brigham and Women’s Hospital and Harvard Medical School, Boston, MA USA; 7 Broad Institute of Massachusetts Institute of Technology and Harvard, Cambridge, MA USA; 8 Department of Oncologic Pathology, Dana-Farber Cancer Institute, Boston, MA USA; 9 Program in MPE Molecular Pathological Epidemiology, Department of Pathology, Brigham and Women’s Hospital, and Harvard Medical School, Boston, MA USA; 10 Department of Epidemiology, Harvard T.H. Chan School of Public Health, Boston, MA USA

**Keywords:** Molecular epidemiology, Premalignancy, Biomarker, Sex difference, Colorectal cancer

## Abstract

**Background:**

Sex hormones have been suggested to play a role in colorectal cancer (CRC), but their influence on early initiation of CRC remains unknown.

**Methods:**

We retrospectively examined the associations with risk of CRC precursors, including conventional adenomas and serrated polyps, for plasma estrone, estradiol, free estradiol, testosterone, free testosterone, sex hormone-binding globulin (SHBG), and the ratio of estradiol to testosterone among 5404 postmenopausal women from the Nurses’ Health Study I and II. Multivariable logistic regression was used to calculate the odds ratio (OR) and 95% confidence intervals (CI). Given multiple testing, *P* < 0.005 was considered statistically significant.

**Results:**

During 20 years of follow-up, we documented 535 conventional adenoma cases and 402 serrated polyp cases. Higher concentrations of SHBG were associated with lower risk of conventional adenomas, particularly advanced adenomas (multivariable OR comparing the highest to the lowest quartile, 0.40, 95% CI 0.24–0.67, *P* for trend < 0.0001). A nominally significant association was found for SHBG with lower risk of large serrated polyps (≥ 10 mm) (OR, 0.47, 95% CI 0.17–1.35, *P* for trend = 0.02) as well as free estradiol and free testosterone with higher risk of conventional adenomas (OR, 1.54, 95% CI 1.02–2.31, *P* for trend = 0.03 and OR, 1.33, 95% CI 0.99–1.78, *P* for trend = 0.03, respectively).

**Conclusions:**

The findings suggest a potential role of sex hormones, particularly SHBG, in early colorectal carcinogenesis.

**Supplementary Information:**

The online version contains supplementary material available at 10.1186/s12916-020-01895-1.

## Background

Colorectal cancer (CRC) is highly heterogeneous and can arise from distinct precursor lesions, including conventional adenomas and serrated polyps [1]. In contrast to the conventional pathway characterized by chromosomal instability, the serrated pathway features *BRAF* mutation, CpG island methylator phenotype, and microsatellite instability [2]. According to the 5th edition of the WHO criteria, serrated polyps include hyperplastic polyp (HP), sessile serrated lesion (SSL), SSL with dysplasia, traditional serrated adenoma (TSA), and unclassified serrated adenoma [3]. The serrated pathway is proposed to mainly originate from HPs and transit to SSLs or TSAs before progression to dysplasia and cancer [4, 5], although there is the potential for in situ development of SSLs [6]. Of particular concern, serrated polyps are flat and difficult to remove by endoscopy and have potential for rapid growth, disproportionately contributing to interval CRCs diagnosed after a negative screening test [7].

CRC incidence is lower in women than men across all ages. Also, it appears that women are more likely to develop serrated polyps than men [8, 9]. While the reasons for these sex differences remain unclear, sex hormones have been postulated as a potential explanation [10]. Exposure to exogenous sex hormones, including oral contraceptives [11, 12] and postmenopausal hormone therapy [13, 14], has been linked to lower risk of CRC in some but not all studies [15, 16]. A recent meta-analysis of cohort studies found that hormone replacement therapy, but not oral contraceptives, was associated with lower risk of conventional adenoma [17]. Of note, due to first-pass metabolism by the liver, exogenous hormones may exert certain biological effects different from those of endogenous estrogens [18].

Current data on endogenous sex hormones and CRC risk are sparse and yield inconsistent results. Two studies reported no association for estrone or estradiol [19, 20], while others found positive [21, 22] or inverse associations for these hormones [23]. Similar inconsistency has been observed for CRC with other components of the sex hormone axis, including testosterone and sex hormone-binding globulin (SHBG).

In women, testosterone is mainly produced by the ovaries (even after menopause), adrenal glands, and peripheral conversion of androgen precursors produced by the adrenals and ovaries [24]. Experimental evidence suggests that testosterone may promote the development of colonic adenoma [25]. SHBG is a glycoprotein synthesized in the liver and regulates sex hormone bioactivity through binding with high affinity to circulating testosterone and estradiol [26]. To our knowledge, no epidemiologic studies have yet examined circulating levels of sex hormones in relation to conventional adenomas or serrated polyps.

Therefore, we performed this retrospective study to evaluate the associations of prediagnostic sex hormones with risk of conventional adenoma and serrated polyp among 5404 postmenopausal women from the Nurses’ Health Study (NHS) I and II. We assessed estrone, estradiol, free estradiol, testosterone, free testosterone, and SHBG, as well as the ratio of estradiol to testosterone, which reflects the activity of aromatase conversion from testosterone to estradiol.

## Methods

### Study population

The NHSI and NHSII are two US prospective cohort studies that included 121,700 registered female nurses aged 30–55 years since 1976 and 116,686 female nurses aged 25–42 years since 1989, respectively. The enrolled women completed a questionnaire at study baseline and were mailed follow-up questionnaires biennially to update their lifestyle and medical information, with cumulative follow-up rates greater than 90% in both cohorts [27]. In NHSI, 32,826 women donated blood samples via overnight courier on ice packs in 1989–1990, and in NHSII, 29,611 provided blood samples using a similar method in 1996–1999. Upon arrival, samples were centrifuged, aliquoted, and stored in liquid nitrogen freezers immediately [28]. Demographic, dietary, and lifestyle profiles of women who provided blood samples were generally similar to those who did not [29].

The current study initially included 8055 NHSI and 4574 NHSII women with available sex hormone data (i.e., estrone, estradiol, testosterone, and SHGB) predominately from previous nested case-control studies of various outcomes conducted between 1997 and 2014 [30]. We excluded premenopausal women and those whose biomarker data were considered as outliers by the generalized extreme studentized deviate many-outlier approach [31] and who had a history of cancer (except non-melanoma skin cancer), colorectal polyp, or inflammatory bowel disease at the time of blood draw, or had no lower gastrointestinal endoscopy after blood donation (Additional file 1: Figure S1 and Table S1). A total of 5404 postmenopausal women were included in the final analysis. Participants showed similar demographic and lifestyle characteristics compared to all postmenopausal women who provided a blood sample in the two cohorts (Additional file 1: Table S2).

The study was approved by the institutional review boards of the Brigham and Women’s Hospital and Harvard T.H. Chan School of Public Health and those of participating registries as required.

### Assessment of circulating biomarkers

Plasma concentrations of estrone, estradiol, and testosterone were measured in the Molecular lab at the Mayo Clinic (Rochester, MN) using the turbulent flow liquid chromatography tandem mass spectrometry. SHBG was assayed in Dr. Rifai’s Lab at the Children’s Hospital (Boston, MA) using a competitive electrochemiluminescence immunoassay. Levels of C-peptide, a marker of insulin secretion, were determined by using enzyme-linked immunosorbent assay in Dr. Pollak’s laboratory at McGill University (Montreal, QC, Canada). Quality control samples were randomly interspersed among the case-control samples. Laboratory personnel were blinded to quality control, case-control status, or any other participant information. The median intra-assay coefficient of variation from quality control samples ranged from 6.8 to 12.0% for the studied biomarkers [30]. To control for variations across batches, we recalibrated hormone concentrations within each cohort to the value of an “average batch” using the method proposed by Rosner and colleagues [32]. Free estradiol and testosterone concentrations were calculated by a validated algorithm based on total estradiol or testosterone, SHBG, an assumed constant for normal albumin concentrations, and affinity constants of SHBG and albumin for estradiol or testosterone [33]. The ratio of total estradiol to total testosterone was also calculated.

### Assessment of covariates

Covariate data were collected through self-administered questionnaires at baseline and during follow-up biennially. In the current study, we used covariate data from the questionnaires closest to blood draw, including body mass index (BMI), smoking status, alcohol consumption, Alternate Healthy Eating Index (AHEI), physical activity, family history of CRC, regular use of aspirin, and postmenopausal hormone use. These covariates are plausible risk factors for colorectal polyps and CRC [34, 35] and might be associated with sex hormone levels [36, 37]. BMI was calculated as body weight in kilograms divided by the square of height in meters. Physical activity was assessed by the product sum of the metabolic equivalent values of each specific recreational activity and hours spent on that activity per week [38]. Positive family history of CRC was defined as report of a CRC diagnosis in a parent or sibling. Consistent with our previous studies [39, 40], regular aspirin use was defined as use of at least two standard-equivalent tablets per week. Alcohol consumption and AHEI were derived from validated food frequency questionnaires. AHEI is a dietary score measuring the adherence to a dietary pattern characterized by foods and nutrients strongly associated with risk of chronic disease [41].

### Ascertainment of colorectal polyps

Ascertainment of colorectal polyps in the NHSI and NHSII has been described in detail previously [35]. Briefly, on each biennial questionnaire, participants were asked whether they had undergone a colonoscopy or sigmoidoscopy and whether any colorectal polyp had been diagnosed in the past 2 years. When a participant reported a polyp diagnosis, written consent was to obtain to review her endoscopic and pathologic records. Study physicians, who were unaware of exposure information, confirmed the diagnosis and extracted data on histology, size, number, and anatomic location of polyps. Conventional adenomas included tubular, tubulovillous and villous adenomas, and adenomas with high-grade dysplasia. Advanced adenomas were defined as at least one adenoma of ≥ 10 mm in diameter or with advanced histology (tubulovillous/villous histological features or high-grade or severe dysplasia) [42]. Serrated polyps comprised HPs and mixed/serrated adenomas. The latter consisted of both mixed polyps (those with both adenomatous and hyperplastic changes in histology) and polyps with any serrated diagnosis (e.g., serrated adenomas, serrated polyps, and sessile serrated adenoma/polyps). If a participant had more than one adenoma or serrated polyp in a sublocation (proximal colon, distal colon, or rectum) in an endoscopy, the histology of the most advanced lesion and the size of the largest polyp were used for that sublocation. Although we did not conduct systematic record review for individuals who reported to have undergone an endoscopy but no polyps, our previous validation study in a random sample of 114 women indicated an accuracy of 97% for self-reported negative endoscopies [43].

### Statistical analysis

The current study only included postmenopausal women who had undergone at least one lower endoscopy since blood draw. If a woman reported more than one endoscopy during the study period, multiple records were used in the analysis. Participants were censored at the time of the first diagnosis of colorectal polyps, death, or the end of follow-up (June 1, 2012, for the NHSI and June 1, 2011, for the NHSII), whichever occurred first. To account for multiple records per participant, we applied an Andersen-Gill data structure with a new record for each 2-year follow-up during which a participant underwent an endoscopy [44].

We log transformed the concentrations of sex hormones to improve normality. Multivariable logistic regression for clustered data (PROC GENMOD) was used to account for repeated observations (i.e., multiple endoscopies) and to compute odds ratios (ORs) of conventional adenoma and serrated polyp, categorically according to quartiles and continuously per one standard deviation (SD) increment in hormone concentrations, with adjustment for potential confounders (plausible risk factors for colorectal polyps and CRC and might be associated with sex hormone levels). *P* for trend was calculated by a Wald test. To maximize the ability for confounding control, we calculated the averages of body mass index, physical activity, alcohol consumption, and AHEI using the two adjacent questionnaires administered most proximately to blood draw. For missing data of covariates on a questionnaire (all less than 2%), we carried forward available information from prior questionnaires. To test for potential non-linearity, we performed restricted cubic spline analyses and used a likelihood ratio test to compare the model with the linear term only to the model with both the linear and spline terms. No strong statistical evidence for non-linearity was found. We also compared the biomarker associations between conventional adenoma and serrated polyp through a case-only analysis and calculated *P* for heterogeneity [45].

We further performed separate analyses according to histopathological features of polyps. Sensitivity analyses were performed among postmenopausal women who were not using hormone therapy and among those who were selected as controls in the source case-control studies. In a subset of participants who also had C-peptide measurements (*n* = 2330), we additionally controlled for C-peptide levels. Moreover, we analyzed the association of sex hormones with polyp risk according to the median time interval since blood draw (< 11 years, ≥ 11 years).

To account for multiple hypothesis testing, we used a stringent α level of 0.005 as recommended by Benjamin et al. to reduce the chance findings or so-called false positives [46]. All statistical tests were two-sided and conducted in SAS version 9.4 software (SAS Institute Inc., Cary, NC).

## Results

During 20 years of follow-up of 5404 postmenopausal women in the NHSI and NHSII, we documented 535 cases of conventional adenoma and 402 cases of serrated polyp, including 109 cases having synchronous conventional adenomas and serrated polyps. The median time interval between blood draw and polyp diagnosis was 11.0 years (interquartile range, 6.7–14.1). As shown in Table 1, compared to women without any polyps, those who developed conventional adenomas or serrated polyps were more likely to have a higher BMI, report a family history of CRC, smoke more cigarettes, drink more alcohol, have a lower AHEI, and were less likely to be physically active at blood draw. A modest/high correlation was found among sex hormones (Additional file 1: Table S3).

**Table 1:** Baseline characteristics of postmenopausal women from the NHSI and NHSII

Variable^a^	Non-polyp	Conventional adenoma	Serrated polyp
No. of participants	4576	535	402
Age at blood draw, years	58.9 (6.1)	59.4 (5.8)	57.5 (6.1)
White, %	99	99	99
Height, cm	164.0 (6.1)	164.6 (5.9)	164.4 (5.0)
Body mass index^b^, kg/m^2^	25.3 (4.5)	25.9 (4.0)	26.1 (4.1)
Family history of colorectal cancer, %	27	34	29
Pack-year of smoking	11.7 (18.0)	15.1 (18.8)	17.8 (18.5)
Never, %	46	44	34
Past, %	44	43	47
Current, %	10	14	20
Alcohol intake^b^, g/day	6.1 (9.6)	6.3 (8.5)	6.4 (8.8)
Physical activity^b, c^, MET-hours/week	19.1 (19.7)	18.7 (19.3)	17.7 (16.5)
AHEI dietary score^b^	54.7 (10.2)	53.4 (9.1)	53.3 (8.9)
Regular aspirin use^d^, %	52	53	49
Biomarker^e^			
Estrone (pg/ml)	24.40 (17.10–35.19)	25.23 (19.11–37.22)	25.31 (18.47–38.71)
Total estradiol (pg/ml)	5.79 (4.06–9.42)	6.02 (4.39–10.58)	6.64 (4.33–11.38)
Free estradiol (pg/ml)	0.08 (0.05–0.13)	0.09 (0.05–0.15)	0.09 (0.05–0.15)
Total testosterone (ng/dl)	18.70 (13.47–25.76)	18.22 (12.93–25.07)	18.09 (13.27–25.85)
Free testosterone (ng/dl)	0.13 (0.08–0.20)	0.14 (0.10–0.23)	0.14 (0.10–0.22)
SHBG (nmol/L)	71.72 (47.57–113.38)	60.70 (40.90–92.75)	60.21 (41.12–93.69)
Total estradiol (pg/ml)/total testosterone (pg/ml)	0.03 (0.02–0.05)	0.03 (0.02–0.06)	0.04 (0.02–0.06)

*Abbreviations*: *NHS* the Nurses’ Health Study, *MET* metabolic equivalent task, *AHEI* Alternative Healthy Eating Index, *SHBG* sex hormone-binding globulin

^a^All variables are standardized by age at blood draw except age. Mean (SD) is presented for continuous variables and percentage for categorical variables, unless otherwise specified

^b^The average calculated using the two adjacent questionnaires most proximate to blood draw

^c^Physical activity is represented by the product sum of the METS of each specific recreational activity and hours spent on that activity per week

^d^A standard tablet contains 325 mg aspirin and regular users were defined as those who used at least two tablets per week

^e^The natural-log transformed biomarker concentrations were back transformed and presented as median values (quartiles). Free estradiol and free testosterone were calculated using a validated algorithm based on total estradiol or total testosterone, SHBG, an assumed constant representing the normal albumin concentration, and the association constants for the binding of estradiol and testosterone to SHBG and albumin

Tables 2 and 3 show the associations of sex hormones with conventional adenomas and serrated polyps, respectively. Higher concentrations of SHBG were associated with lower risk of conventional adenomas (*P* for trend < 0.0001), with the multivariable OR of 0.47 (95% CI 0.35–0.64) comparing the highest to the lowest quartile. A suggestive inverse association was also found for SHBG with serrated polyps (OR, 0.72, 95% CI 0.51–1.02, *P* for trend = 0.01). By contrast, higher levels of free estradiol and free testosterone were nominally associated with increased risk of conventional adenomas (OR, 1.54, 95% CI 1.02–2.31, *P* for trend = 0.03 and OR, 1.33, 95% CI 0.99–1.78, *P* for trend = 0.03, respectively). No association was found for estrone, total estradiol, total testosterone, or the ratio of total estradiol to testosterone.

**Table 2:** Associations between plasma sex hormones and conventional adenoma in postmenopausal women from the NHSI and NHSII

Biomarker	Conventional adenoma, OR (95% CI)^a^					
	Q1 (lowest)	Q2	Q3	Q4 (highest)	*P* for trend	Per 1-SD^b^
Estrone						
No. of cases	63	78	70	74		
Model 1	1	1.30 (0.93–1.84)	1.20 (0.84–1.71)	1.39 (0.98–1.99)	0.12	1.10 (0.98–1.24)
Model 2	1	1.32 (0.93–1.87)	1.20 (0.83–1.72)	1.35 (0.92–2.00)	0.19	1.09 (0.96–1.25)
Total estradiol						
No. of cases	73	81	68	84		
Model 1	1	1.15 (0.83–1.59)	0.98 (0.69–1.37)	1.41 (1.01–1.96)	0.06	1.15 (0.99–1.33)
Model 2	1	1.14 (0.82–1.59)	0.98 (0.68–1.40)	1.39 (0.96–2.01)	0.09	1.16 (0.98–1.38)
Free estradiol						
No. of cases	67	63	66	81		
Model 1	1	0.94 (0.66–1.33)	1.04 (0.73–1.48)	1.48 (1.05–2.09)	0.01	1.21 (1.04–1.41)
Model 2	1	0.93 (0.65–1.33)	1.10 (0.76–1.58)	1.54 (1.02–2.31)	0.03	1.23 (1.02–1.47)
Total testosterone						
No. of cases	139	119	118	110		
Model 1	1	0.88 (0.68–1.13)	0.85 (0.66–1.09)	0.84 (0.64–1.09)	0.34	0.96 (0.87–1.05)
Model 2	1	0.88 (0.68–1.13)	0.83 (0.64–1.07)	0.82 (0.62–1.07)	0.26	0.95 (0.86–1.04)
Free testosterone						
No. of cases	94	116	110	129		
Model 1	1	1.26 (0.95–1.66)	1.23 (0.92–1.62)	1.56 (1.19–2.05)	0.001	1.19 (1.08–1.32)
Model 2	1	1.18 (0.89–1.56)	1.09 (0.82–1.46	1.33 (0.99–1.78)	0.03	1.13 (1.01–1.25)
SHBG						
No. of cases	158	128	125	83		
Model 1	1	0.73 (0.58–0.94)	0.71 (0.56–0.91)	0.45 (0.34–0.60)	<.0001	0.77 (0.70–0.84)
Model 2	1	0.74 (0.58–0.96)	0.73 (0.56–0.94)	0.47 (0.35–0.64)	<.0001	0.78 (0.70–0.86)
Total estradiol/total testosterone						
No. of cases	67	70	74	74		
Model 1	1	1.04 (0.73–1.47)	1.10 (0.78–1.55)	1.24 (0.87–1.75)	0.26	1.08 (0.94–1.25)
Model 2	1	1.05 (0.73–1.50)	1.07 (0.74–1.54)	1.20 (0.81–1.76)	0.43	1.07 (0.91–1.26)

*Abbreviations*: *NHS* the Nurses’ Health Study, *OR* odds ratio, *CI* confidence interval, *SHBG* sex hormone-binding globulin, *MET* metabolic equivalent task, *AHEI* Alternative Healthy Eating Index

^a^Model 1 was adjusted for age (continuous), case or control status, fasting status (yes or no), time period of endoscopy (in 2-year intervals), number of prior endoscopies (continuous), and time in years since the most recent endoscopy (continuous); model 2 was additionally adjusted for race (Caucasian or non-Caucasian), family history of colorectal cancer (yes or no), height (continuous), smoking status (never, ever, or current), AHEI score (quartile), body mass index (continuous), physical activity (< 3.0, 3.0–8.9, 9.0–17.9, 18.0–26.9, ≥ 27.0 MET-hours/week), alcohol consumption (0, 0.1–4.9, 5.0–9.9, 10.0–14.9, ≥ 15.0 g/day), regular aspirin use (yes or no), and postmenopausal hormone therapy (never, ever, or current)

^b^SD was the standard deviation of log-transformed hormone levels: 0.58 for estrone, 0.79 for total estradiol, 0.84 for free estradiol, 0.50 for total testosterone, 0.64 for free testosterone, 0.62 for SHBG, and 0.80 for the ratio of total estradiol to total testosterone

**Table 3:** Associations between plasma sex hormones and serrated polyp in postmenopausal women from the NHSI and NHSII

Biomarker	Serrated polyp, OR (95% CI)^a^					
	Q1 (lowest)	Q2	Q3	Q4 (highest)	*P* for trend	Per 1-SD^b^
Estrone						
No. of cases	45	48	47	53		
Model 1	1	1.13 (0.74–1.71)	1.11 (0.73–1.69)	1.39 (0.92–2.11)	0.33	1.07 (0.93–1.23)
Model 2	1	1.11 (0.72–1.69)	1.05 (0.69–1.61)	1.26 (0.79–1.99)	0.74	1.03 (0.88–1.21)
Total estradiol						
No. of cases	52	47	48	65		
Model 1	1	0.93 (0.62–1.39)	0.96 (0.64–1.43)	1.51 (1.04–2.19)	0.02	1.24 (1.04–1.47)
Model 2	1	0.90 (0.59–1.35)	0.86 (0.57–1.30)	1.30 (0.85–1.99)	0.15	1.17 (0.95–1.44)
Free estradiol						
No. of cases	47	38	54	55		
Model 1	1	0.77 (0.50–1.19)	1.20 (0.81–1.80)	1.40 (0.94–2.10)	0.01	1.28 (1.07–1.52)
Model 2	1	0.69 (0.44–1.08)	1.09 (0.72–1.65)	1.14 (0.70–1.86)	0.13	1.18 (0.95–1.46)
Total testosterone						
No. of cases	96	96	88	89		
Model 1	1	1.02 (0.76–1.36)	0.90 (0.67–1.21)	0.96 (0.71–1.29)	0.66	0.98 (0.88–1.09)
Model 2	1	1.01 (0.76–1.36)	0.86 (0.64–1.16)	0.88 (0.65–1.20)	0.32	0.95 (0.85–1.05)
Free testosterone						
No. of cases	75	86	93	92		
Model 1	1	1.17 (0.85–1.60)	1.34 (0.99–1.83)	1.47 (1.08–2.01)	0.003	1.19 (1.06–1.34)
Model 2	1	1.04 (0.76–1.43)	1.05 (0.76–1.45)	1.03 (0.73–1.44)	0.49	1.05 (0.92–1.19)
SHBG						
No. of cases	117	102	78	81		
Model 1	1	0.79 (0.60–1.04)	0.59 (0.44–0.79)	0.55 (0.41–0.73)	<.0001	0.79 (0.71–0.87)
Model 2	1	0.89 (0.67–1.18)	0.70 (0.51–0.96)	0.72 (0.51–1.02)	0.01	0.86 (0.76–0.97)
Total estradiol/total testosterone						
No. of cases	48	45	46	59		
Model 1	1	0.91 (0.60–1.38)	0.95 (0.63–1.43)	1.35 (0.91–2.01)	0.08	1.16 (0.98–1.38)
Model 2	1	0.86 (0.56–1.32)	0.86 (0.56–1.32)	1.23 (0.78–1.92)	0.24	1.13 (0.92–1.38)

*Abbreviations*: *NHS* the Nurses’ Health Study, *OR* odds ratio, *CI* confidence interval, *SHBG* sex hormone-binding globulin, *MET* metabolic equivalent task, *AHEI* Alternative Healthy Eating Index

^a^Model 1 was adjusted for age (continuous), case or control status, fasting status (yes or no), time period of endoscopy (in 2-year intervals), number of prior endoscopies (continuous), and time in years since the most recent endoscopy (continuous); model 2 was additionally adjusted for race (Caucasian or non-Caucasian), family history of colorectal cancer (yes or no), height (continuous), smoking status (never, ever, or current), AHEI score (quartile), body mass index (continuous), physical activity (< 3.0, 3.0–8.9, 9.0–17.9, 18.0–26.9, ≥ 27.0 MET-hours/week), alcohol consumption (0, 0.1–4.9, 5.0–9.9, 10.0–14.9, ≥ 15.0 g/day), regular aspirin use (yes or no), and postmenopausal hormone therapy (never, ever, or current)

^b^SD was the standard deviation of log-transformed hormone levels: 0.58 for estrone, 0.79 for total estradiol, 0.84 for free estradiol, 0.50 for total testosterone, 0.64 for free testosterone, 0.62 for SHBG, and 0.80 for the ratio of total estradiol to total testosterone

Given the established malignant potential of advanced conventional adenoma and large serrated polyp, we then focused on these high-risk lesions and examined their associations with sex hormones (Table 4). SHBG was inversely associated with risk of advanced conventional adenomas (*P* for trend < 0.0001), with the OR comparing extreme quartiles of 0.40 (95% CI 0.24–0.67). A nominally significant association was also observed for SHBG and lower risk of large serrated polyps (OR, 0.47, 95% CI 0.17–1.35, *P* for trend = 0.02). In addition, higher concentrations of free testosterone were suggestively associated with an increased risk of advanced conventional adenomas (OR, 1.72, 95% CI 1.08–2.73, *P* for trend = 0.02).

**Table 4:** Associations of plasma sex hormones with advanced conventional adenoma and large serrated polyp in postmenopausal women from the NHSI and NHSII

Biomarker	Q1 (lowest)	Q2	Q3	Q4 (highest)	*P* for trend	Per 1-SD^b^
Advanced conventional adenoma (*N* = 208), OR (95% CI)^a^						
Estrone	1	2.10 (1.16–3.83)	2.10 (1.13–3.90)	1.75 (0.89–3.45)	0.09	1.19 (0.97–1.45)
Total estradiol	1	1.20 (0.71–2.03)	1.12 (0.63–1.97)	1.29 (0.70–2.36	0.24	1.18 (0.90–1.54)
Free estradiol	1	0.77 (0.43–1.38)	1.29 (0.74–2.23)	1.36 (0.72–2.57)	0.20	1.20 (0.91–1.59)
Total testosterone	1	0.66 (0.43–1.01)	0.82 (0.56–1.22)	0.78 (0.52–1.17)	0.72	0.97 (0.84–1.13)
Free testosterone	1	1.25 (0.78–2.00)	1.24 (0.76–2.01)	1.72 (1.08–2.73)	0.02	1.24 (1.03–1.48)
SHBG	1	0.85 (0.58–1.24)	0.66 (0.43–0.99)	0.40 (0.24–0.67)	< .0001	0.71 (0.61–0.83)
Total estradiol/total testosterone	1	1.21 (0.70–2.10)	1.13 (0.64–2.00)	1.00 (0.53–1.88)	0.94	0.99 (0.78–1.26)
Large serrated polyp (≥ 10 mm) (*N* = 34), OR (95% CI)^a^						
Estrone	1	0.87 (0.19–3.90)	0.92 (0.22–3.86)	1.77 (0.48–6.54)	0.48	1.19 (0.74–1.93)
Total estradiol	1	0.66 (0.15–2.81)	0.69 (0.15–3.18)	1.64 (0.50–5.45)	0.51	1.17 (0.74–1.85)
Free estradiol	1	0.29 (0.06–1.45)	0.66 (0.16–2.70)	1.48 (0.38–5.76)	0.38	1.24 (0.77–2.02)
Total testosterone	1	0.65 (0.23–1.82)	0.62 (0.22–1.77)	0.92 (0.35–2.42)	0.65	0.91 (0.62–1.35)
Free testosterone	1	0.65 (0.23–1.86)	0.74 (0.24–2.24)	0.80 (0.28–2.32)	0.79	1.06 (0.71–1.58)
SHBG	1	0.63 (0.26–1.52)	0.34 (0.11–1.02)	0.47 (0.17–1.35)	0.02	0.87 (0.77–0.98)
Total estradiol/total testosterone	1	0.98 (0.23–4.12)	0.53 (0.11–2.58)	1.11 (0.28–4.41)	0.93	0.98 (0.58–1.65)

*Abbreviations: NHS* the Nurses’ Health Study, *OR* odds ratio, *CI* confidence interval, *SHBG* sex hormone-binding globulin, *MET* metabolic equivalent task, *AHEI* Alternative Healthy Eating Index

^a^Adjusted for age (continuous), case or control status, fasting status (yes or no), time period of endoscopy (in 2-year intervals), number of prior endoscopies (continuous), and time in years since the most recent endoscopy (continuous), race (Caucasian or non-Caucasian), family history of colorectal cancer (yes or no), height (continuous), smoking status (never, ever, or current), AHEI score (quartile), body mass index (continuous), physical activity (< 3.0, 3.0–8.9, 9.0–17.9, 18.0–26.9, ≥ 27.0 MET-hours/week), alcohol consumption (0, 0.1–4.9, 5.0–9.9, 10.0–14.9, ≥ 15.0 g/day), regular aspirin use (yes or no), and postmenopausal hormone therapy (never, ever, or current). Participants with non-advanced lesions and no polyps were treated as the reference

^b^SD was the standard deviation of log-transformed hormone levels: 0.58 for estrone, 0.79 for total estradiol, 0.84 for free estradiol, 0.50 for total testosterone, 0.64 for free testosterone, 0.62 for SHBG, and 0.80 for the ratio of total estradiol to total testosterone

The associations remained essentially unchanged when we restricted the analysis to postmenopausal women who were not taking hormone therapy at the time of blood draw (Additional file 1: Table S4) or control participants of the original case-control studies (Additional file 1: Table S5). When additionally adjusting for C-peptide levels in model 2, the associations of free estradiol and testosterone with conventional adenoma were attenuated to null (*P* for trend = 0.42 and 0.27, respectively), while the associations of SHBG with conventional adenoma and serrated polyp remained essentially unchanged (*P* for trend = 0.0003 and 0.01, respectively) (Additional file 1: Table S6). In the stratified analysis by the time interval since blood draw, the inverse association between SHBG and adenoma was statistically significant in both subgroups (< and ≥ 11 years) (Additional file 1: Table S7).

## Discussion

In this retrospective analysis of 5404 postmenopausal women in the NHS cohorts, we found that higher concentrations of SHBG were associated with lower risk of conventional adenomas, particularly advanced adenomas. In addition, a suggestive association was found for SHBG with lower risk of large serrated polyps and free estradiol and free testosterone with higher risk of conventional adenomas. These data indicate the potential roles of sex hormones in the early stages of colorectal carcinogenesis.

Several epidemiologic studies have evaluated the association between circulating levels of SHBG and CRC risk. A case-control study nested in 4 US prospective cohorts showed an inverse association between plasma SHBG and CRC risk in men even after adjusting for BMI and C-peptide, but no association in postmenopausal women [19]. Additional two studies of postmenopausal women reported null results [20, 22]; however, the numbers of CRC patients in these studies were relatively small (*n* < 200) and the statistical power might be limited. In contrast, an investigation of 401 postmenopausal women with CRC enrolled in the Women’s Health Initiative Clinical Trial found a positive association between SHBG levels and CRC risk [23]. The reasons for the conflicting results remain unknown and may relate to the differences in the study population, assaying methods, and covariate adjustments [23]. In line with the free hormone hypothesis, the current study showed an inverse correlation between SHBG and free estradiol and testosterone. Furthermore, we found that higher SHBG levels were associated with lower risk of conventional adenomas, particularly advanced lesions. In addition to modulating sex hormone balance, in vitro evidence suggests that SHBG may suppresses inflammation and lipid accumulation in macrophages and adipocytes independent of testosterone and estradiol [47]. Besides, observational and Mendelian randomization studies have consistently linked lower SHBG levels to an increased risk of insulin resistance and type 2 diabetes, which are important risk factors for CRC, supporting the beneficial role of SHBG in mitigating metabolic perturbations [48, 49]. The observed associations for SHBG in our study remained robust to adjustment for both BMI and C-peptide, suggesting an independent role of SHBG in early colorectal carcinogenesis. Further mechanistic investigations are warranted.

In support of our findings for free estradiol and conventional adenoma, two independent studies have reported positive associations between endogenous levels of estrogens and CRC risk in postmenopausal women [21, 22]. However, these findings are inconsistent with three other studies, including two with null results [19, 20] and one reporting inverse associations for total/free estradiol and estrone [23]. The reasons for the divergent results may be partly attributed to the different assay methods. It has been noted that radioimmunoassay in prior studies is less specific than liquid chromatography tandem mass spectrometry and therefore the levels of estrogens measured by radioimmunoassay are likely to be overestimated [22, 23], while electrochemiluminescence may not be able to detect the very low levels in postmenopausal women [20]. Mechanistically, evidence from in vitro and in vivo studies, although somewhat inconsistent, has suggested the mitogenic and tumorigenic effects of estradiol on colorectal cells [50, 51]. In our study, further adjustment for C-peptide attenuated the association of free estradiol with conventional adenoma, suggesting that the effects may be related to insulin resistance. There is a perception that high levels of circulating estradiol is an indicator of estrogen resistance concomitant with insulin resistance [52].

Regarding endogenous testosterone, a nested case-control study of Japanese postmenopausal women showed a positive association between plasma total testosterone and CRC [20]. Moreover, a joint analysis of the NHS and Women’s Health Study found an OR of 1.43 for CRC (95% CI 0.82–2.50) comparing postmenopausal women in the highest quartile of total testosterone to those in the lowest quartile [19]. Since most testosterone is bound to SHBG or albumin, the free portion (less than 2%) is hypothesized to be most biologically active [53].We observed a suggestive positive association between free testosterone and risk of total and advanced conventional adenoma, supporting a pro-CRC effect of testosterone in postmenopausal women. Although the biological mechanisms remain unclear, testosterone has been shown to stimulate growth of colon cancer cells in vitro and the effect can be inhibited by anti-androgens [54]. Evidence from animal models also indicates that testosterone may promote formation of colorectal adenomas [25]. Additionally, testosterone can exert adverse metabolic effects in women by influencing fat mass expansion and distribution, insulin signaling, and lipid metabolism [55]. Given the established role of adiposity in CRC [56, 57], testosterone may also promote CRC through its metabolic effects.

The major strengths of our study include long-term follow-up and detailed data on covariates that allow for robust confounding control. Moreover, we ascertained polyp diagnosis through medical record review and collected detailed histopathologic information of both conventional adenomas and serrated polyps. Several limitations also should be acknowledged. First, because of the evolving nature and lack of consensus on the diagnostic criteria for specific subtypes of serrated polyps, HPs were difficult to separate from SSLs and TSAs in pathologic records. However, prior data showed that serrated polyps ≥ 10 mm in size, which were likely SSLs, were strongly associated with CRC [58]. Second, although existing evidence indicates that sex hormones are generally stable over at least a 4-year period [59, 60], the current study had only a one-time blood measure for each participant, which may not well reflect a longer period of exposure. Third, our study participants were all female health professionals and almost entirely whites. Further investigations in men and racially diverse populations are needed.

## Conclusions

We observed an inverse association of SHBG with risk of overall conventional adenomas and advanced adenomas, as well as a suggestive positive association for free estradiol and free testosterone. Our findings indicate a potential role of sex hormones in early stages of colorectal carcinogenesis. Further studies are needed to confirm our findings and assess the clinical utility of sex hormones for CRC risk stratification.

## Supplementary Information

**Additional file 1: Figure S1.** Flowchart of study participant selection. **Table S1.** Cases and controls included in this study by their original study status in the NHSI and NHSII. **Table S2.** Baseline characteristics of all postmenopausal women at blood draw and those included in the current study. **Table S3.** Age-adjusted Spearman correlation coefficients between plasma sex hormones and body mass index (BMI) in postmenopausal women from the NHSI and NHSII. **Table S4.** Associations of plasma sex hormones with conventional adenoma, serrated polyp, and advanced lesions in postmenopausal women not taking hormone therapy at blood draw from the NHSI and NHSII. **Table S5.** Associations of plasma sex hormones with conventional adenoma and serrated polyp in postmenopausal women who were selected as controls in the source case-control studies from the NHSI and NHSII. **Table S6.** Associations of plasma sex hormones with conventional adenoma and serrated polyp after further adjustment for plasma C-peptide levels in postmenopausal women from the NHSI and NHSII. **Table S7.** Associations of plasma sex hormones with conventional adenoma and serrated polyp in postmenopausal women according to the median time interval since blood draw.

## Data Availability

The NHSI and NHSII datasets can be made available upon reasonable request for scientific collaborations. Further information including the procedures to obtain and access data from the Nurses’ Health Studies is described at https://www.nurseshealthstudy.org/researchers (contact email: nhsaccess@channing.harvard.edu).

## Abbreviations

AHEI: Alternate Healthy Eating Index
BMI: Body mass index
CI: Confidence interval
CRC: Colorectal cancer
HP: Hyperplastic polyp
MET: Metabolic equivalent of task
NHS: The Nurses’ Health Study
OR: Odds ratio
SD: Standard deviation
SHBG: Sex hormone-binding globulin
SSL: Sessile serrated lesion
