# Supplementary material for: Plasma sex hormones and risk of conventional and serrated precursors of colorectal cancer in postmenopausal women
Source: BMC Med. 2021 Jan 28;19:18. doi: 10.1186/s12916-020-01895-1 (PMC7841996; doi:10.1186/s12916-020-01895-1)
Supplement: Supplementary file 1 — Additional file 1: Figure S1. Flowchart of study participant selection. Table S1. Cases and controls included in this study by their original study status in the NHSI and NHSII. Table S2. Baseline characteristics of all postmenopausal women at blood draw and those included in the current study. Table S3. Age-adjusted Spearman correlation coefficients between plasma sex hormones and body mass index (BMI) in postmenopausal women from the NHSI and NHSII. Table S4. Associations of plasma sex hormones with conventional adenoma, serrated polyp, and advanced lesions in postmenopausal women not taking hormone therapy at blood draw from the NHSI and NHSII. Table S5. Associations of plasma sex hormones with conventional adenoma and serrated polyp in postmenopausal women who were selected as controls in the source case-control studies from the NHSI and NHSII. Table S6. Associations of plasma sex hormones with conventional adenoma and serrated polyp after further adjustment for plasma C-peptide levels in postmenopausal women from the NHSI and NHSII. Table S7. Associations of plasma sex hormones with conventional adenoma and serrated polyp in postmenopausal women according to the median time interval since blood draw. [file 12916_2020_1895_MOESM1_ESM.docx]

**Combined additional file material**

Additional file 1: Figure S1. Flowchart of study participant selection.

Additional file 1: Table S1. Cases and controls included in this study by their original study status in the NHSI and NHSII.

Additional file 1: Table S2. Baseline characteristics of all postmenopausal women at blood draw and those included in the current study.

Additional file 1: Table S3. Age-adjusted Spearman correlation coefficients between plasma sex hormones and body mass index (BMI) in postmenopausal women from the NHSI and NHSII.

Additional file 1: Table S4. Associations of plasma sex hormones with conventional adenoma, serrated polyp, and advanced lesions in postmenopausal women not taking hormone therapy at blood draw from the NHSI and NHSII.

Additional file 1: Table S5. Associations of plasma sex hormones with conventional adenoma and serrated polyp in postmenopausal women who were selected as controls in the source case-control studies from the NHSI and NHSII.

Additional file 1: Table S6. Associations of plasma sex hormones with conventional adenoma and serrated polyp after further adjustment for plasma C-peptide levels in postmenopausal women from the NHSI and NHSII.

Additional file 1: Table S7. Associations of plasma sex hormones with conventional adenoma and serrated polyp in postmenopausal women according to the median time interval since blood draw.

Figure S1. Flowchart of study participant selection.


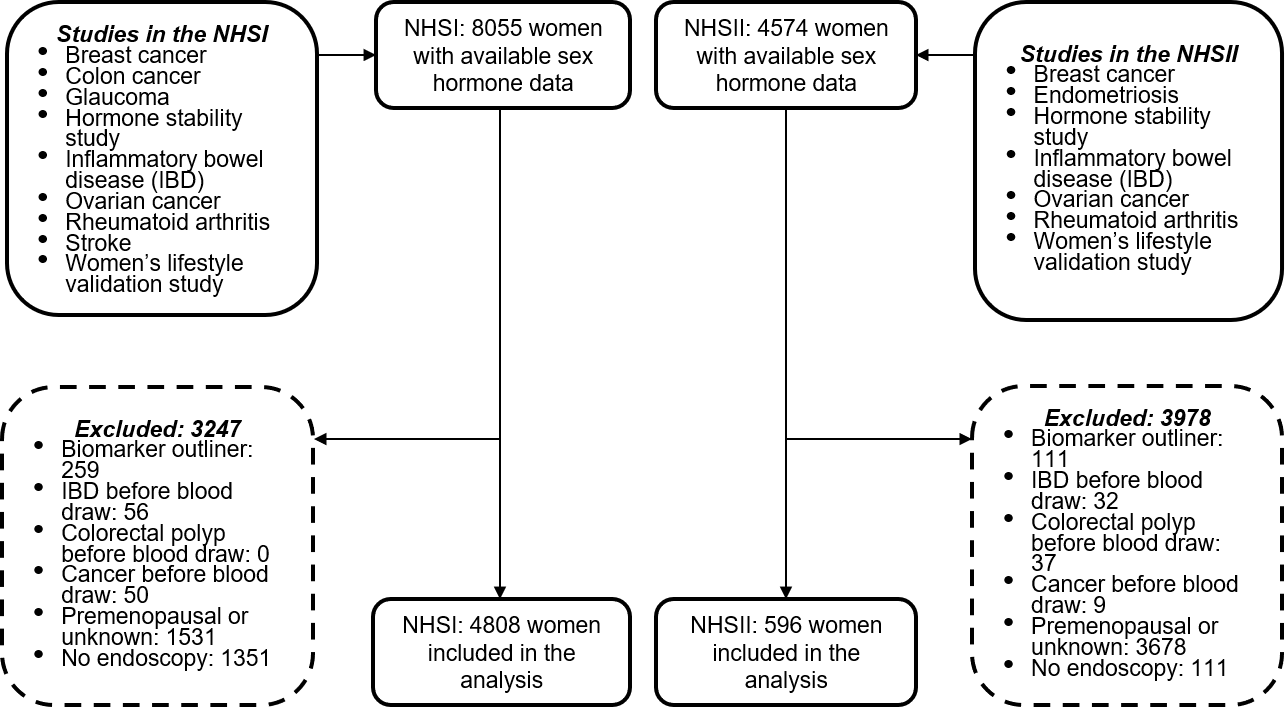


| Table S1. Cases and controls included in this study by their original study status in the NHSI and NHSII | | | | | |  |
| --- | --- | --- | --- | --- | --- | --- |
| Original study | | NHSI | |  | NHSII | |
|  |  | Case | Control |  | Case | Control |
| Breast cancer | | 1191 | 1721 |  | 147 | 239 |
| Colon cancer | | 219 | 251 |  | - | - |
| Endometriosis | | - | - |  | 61 | 98 |
| Glaucoma | | 117 | 117 |  | - | - |
| Hormone stability study^a^ | | - | 56 |  | - | - |
| Inflammatory bowel disease | | 62 | 106 |  | 7 | 17 |
| Ovarian cancer | | 45 | 146 |  | - | - |
| Rheumatoid arthritis | | 49 | 184 |  | 8 | 19 |
| Stroke | | 231 | 241 |  | - | - |
| Women's lifestyle validation study^a^ | | - | 72 |  | - | - |
| Total | | 1914 | 2894 |  | 223 | 373 |
| Abbreviations: NHS, the Nurses’ Health Study.  ^a^ Cross-sectional design. | | | | | | |

| Table S2. Baseline characteristics of all postmenopausal women at blood draw and those included in the current study | | | | | |  |
| --- | --- | --- | --- | --- | --- | --- |
| Variable^a^ | | NHSI | |  | NHSII | |
|  |  | All (n=23132) | Included (n=4808) |  | All (n=5438) | Included (n=596) |
| Age at blood draw, year | | 59.7(5.7) | 60.1(5.4) |  | 47.8(3.5) | 48.0(3.4) |
| White, % | | 98 | 99 |  | 97 | 98 |
| Height, cm | | 163.9(6.1) | 164.0(6.1) |  | 164.8(6.5) | 164.9(6.3) |
| Body mass index, kg/m^2^ | | 25.5(4.6) | 25.4(4.4) |  | 26.8(6.0) | 26.6(5.9) |
| Family history of colorectal cancer, % | | 22 | 24 |  | 15 | 16 |
| Pack-year of smoking | | 13.6(19.8) | 13.0(19.2) |  | 6.5(11.1) | 6.8(11.7) |
| Never, % | | 44 | 45 |  | 60 | 60 |
| Past, % | | 42 | 44 |  | 30 | 30 |
| Current, % | | 13 | 12 |  | 10 | 10 |
| Alcohol intake, g/day | | 5.9(9.7) | 6.1(9.5) |  | 3.6(6.4) | 4.2(7.9) |
| Physical activity^b^, MET-hours/week | | 18.0(18.7) | 18.7(20.7) |  | 18.5(19.6) | 17.5(16.7) |
| AHEI dietary score | | 53.9(10.1) | 54.2(10.2) |  | 51.7(10.4) | 52.2(10.2) |
| Regular aspirin use^c^, % | | 53 | 53 |  | 34 | 35 |
| Abbreviations: NHS, the Nurses’ Health Study; MET, metabolic equivalent task; AHEI, Alternative Healthy Eating Index.  ^a^ All variables are standardized by age at blood draw except age. Mean (SD) is presented for continuous variables and percentage for categorical variables, unless otherwise specified. | | | | | | |
| ^b^ Physical activity is represented by the product sum of the METS of each specific recreational activity and hours spent on that activity per week. | | | | | | |
| ^c^ A standard tablet contains 325 mg aspirin and regular users were defined as those who used at least two tablets per week. | | | | | | |

| Table S3. Age-adjusted Spearman correlation coefficients between plasma sex hormones and body mass index (BMI) in postmenopausal women from the NHSI and NHSII | | | | | | | | | |
| --- | --- | --- | --- | --- | --- | --- | --- | --- | --- |
| Biomarker | Estrone | Total estradiol | Free estradiol | Total testosterone | Free testosterone | SHBG | Total estradiol /total testosterone | C-peptide | BMI |
| Estrone | **–** |  |  |  |  |  |  |  |  |
| Total estradiol | 0.84* | **–** |  |  |  |  |  |  |  |
| Free estradiol | 0.70* | 0.92* | **–** |  |  |  |  |  |  |
| Total testosterone | 0.38* | 0.34* | 0.23* | **–** |  |  |  |  |  |
| Free testosterone | 0.29* | 0.34* | 0.49* | 0.62* | **–** |  |  |  |  |
| SHBG | 0.02 | -0.11* | -0.43* | 0.16* | -0.64* | – |  |  |  |
| Total estradiol /total testosterone | 0.48* | 0.67* | 0.70* | -0.40* | -0.18* | -0.21* | – |  |  |
| C-peptide | 0.10* | 0.22* | 0.35* | -0.13* | 0.21* | -0.40* | 0.28* | – |  |
| BMI | 0.20* | 0.35* | 0.43* | -0.03^#^ | 0.26* | -0.38* | 0.33* | 0.43* | – |
| Abbreviations: NHS, the Nurses’ Health Study; SHBG, sex hormone-binding globulin.  **P* < 0.001; ^#^*P* < 0.05. | | | | | | | | | |

| Table S4. Associations of plasma sex hormones with conventional adenoma, serrated polyp, and advanced lesions in postmenopausal women not taking hormone therapy at blood draw from the NHSI and NHSII | | | | | | |
| --- | --- | --- | --- | --- | --- | --- |
| Biomarker | Q1 (lowest) | Q2 | Q3 | Q4 (highest) | *P* for trend | Per 1-SD^b^ |
| Conventional adenoma (N=329), OR (95% CI)^a^ |  |  |  |  |  |  |
| Estrone | 1 | 1.33(0.93-1.92) | 1.25(0.85-1.84) | 1.40(0.92-2.14) | 0.21 | 1.10(0.95-1.28) |
| Total estradiol | 1 | 1.09(0.77-1.54) | 1.04(0.71-1.52) | 1.36(0.89-2.08) | 0.21 | 1.12(0.94-1.32) |
| Free estradiol | 1 | 1.03(0.70-1.52) | 1.07(0.71-1.61) | 1.66(1.05-2.63) | 0.02 | 1.24(1.03-1.48) |
| Total testosterone | 1 | 0.81(0.57-1.13) | 0.93(0.67-1.28) | 1.01(0.72-1.41) | 0.73 | 1.02(0.91-1.15) |
| Free testosterone | 1 | 1.73(1.08-2.76) | 1.57(0.99-2.48) | 1.81(1.15-2.85) | 0.003 | 1.25(1.08-1.45) |
| SHBG | 1 | 0.73(0.55-0.98) | 0.67(0.49-0.93) | 0.32(0.17-0.59) | <.0001 | 0.73(0.63-0.84) |
| Total estradiol/total testosterone | 1 | 1.09(0.75-1.59) | 1.11(0.76-1.64) | 1.08(0.69-1.71) | 0.71 | 1.04(0.86-1.25) |
| Advanced conventional adenoma (N=129), OR (95% CI)^a^ |  |  |  |  |  |  |
| Estrone | 1 | 2.14(1.14-4.01) | 2.38(1.24-4.58) | 1.90(0.90-4.00) | 0.03 | 1.29(1.03-1.62) |
| Total estradiol | 1 | 1.33(0.76-2.32) | 1.42(0.79-2.57) | 1.47(0.74-2.93) | 0.16 | 1.20(0.93-1.56) |
| Free estradiol | 1 | 0.89(0.48-1.65) | 1.59(0.88-2.88) | 1.45(0.70-3.02) | 0.10 | 1.26(0.96-1.64) |
| Total testosterone | 1 | 0.49(0.26-0.89) | 0.90(0.55-1.46) | 0.95(0.58-1.56) | 0.46 | 1.07(0.89-1.30) |
| Free testosterone | 1 | 1.78(0.82-3.86) | 1.39(0.65-2.97) | 2.03(0.98-4.23) | 0.01 | 1.37(1.08-1.73) |
| SHBG | 1 | 0.79(0.51-1.23) | 0.60(0.36-1.01) | 0.39(0.16-0.97) | 0.001 | 0.68(0.54-0.85) |
| Total estradiol/total testosterone | 1 | 1.31(0.74-2.34) | 1.34(0.74-2.43) | 0.94(0.44-2.02) | 0.87 | 1.02(0.78-1.35) |
| Serrated polyp (N=235), OR (95% CI)^a^ |  |  |  |  |  |  |
| Estrone | 1 | 1.02(0.66-1.59) | 0.90(0.57-1.43) | 1.22(0.74-2.00) | 0.83 | 1.02(0.85-1.23) |
| Total estradiol | 1 | 0.99(0.64-1.52) | 0.85(0.54-1.35) | 1.26(0.77-2.08) | 0.66 | 1.05(0.86-1.27) |
| Free estradiol | 1 | 0.77(0.47-1.26) | 1.06(0.66-1.70) | 0.96(0.53-1.74) | 0.38 | 1.10(0.89-1.36) |
| Total testosterone | 1 | 0.89(0.60-1.33) | 0.95(0.65-1.40) | 0.95(0.64-1.41) | 0.77 | 0.98(0.85-1.13) |
| Free testosterone | 1 | 1.25(0.75-2.09) | 1.23(0.75-2.02) | 1.22(0.74-2.01) | 0.32 | 1.09(0.91-1.31) |
| SHBG | 1 | 0.88(0.63-1.24) | 0.72(0.49-1.08) | 0.83(0.50-1.38) | 0.04 | 0.83(0.70-0.99) |
| Total estradiol/total testosterone | 1 | 0.86(0.55-1.36) | 0.87(0.55-1.39) | 1.12(0.65-1.93) | 0.97 | 1.00(0.81-1.25) |
| Large serrated polyp (≥10 mm) (N=17), OR (95% CI)^a^ |  |  |  |  |  |  |
| Estrone | 1 | 0.58(0.10-3.50) | 0.60(0.13-2.80) | 1.97(0.46-8.43) | 0.56 | 1.20(0.65-2.21) |
| Total estradiol | 1 | 1.21(0.25-5.86) | 0.81(0.12-5.58) | 3.29(0.64-16.81) | 0.13 | 1.52(0.89-2.60) |
| Free estradiol | 1 | 0.64(0.10-4.04) | 1.50(0.27-8.16) | 2.41(0.43-13.61) | 0.04 | 1.76(1.04-2.98) |
| Total testosterone | 1 | 2.35(0.43-12.82) | 1.07(0.16-6.97) | 4.09(0.84-20.00) | 0.06 | 1.45(0.98-2.15) |
| Free testosterone | 1 | 1.40(0.10-20.41) | 2.75(0.24-31.47) | 3.63(0.37-35.36) | 0.01 | 1.85(1.14-2.99) |
| SHBG | 1 | 1.03(0.34-3.13) | 0.31(0.06-1.59) | 0.28(0.03-2.88) | 0.23 | 0.73(0.44-1.22) |
| Total estradiol/total testosterone | 1 | 0.54(0.09-3.25) | 0.56(0.09-3.58) | 1.09(0.18-6.40) | 0.78 | 0.89(0.40-1.99) |
| Abbreviations: NHS, the Nurses’ Health Study; OR, odd ratio; CI, confidence interval; SHBG, sex hormone-binding globulin; MET, metabolic equivalent task; AHEI, Alternative Healthy Eating Index.  ^a^ Adjusted for age (continuous), case or control status, fasting status (yes or no), time period of endoscopy (in 2-year intervals), number of prior endoscopies (continuous), time in years since the most recent endoscopy (continuous), race (Caucasian or non-Caucasian), family history of colorectal cancer (yes or no), height (continuous), smoking status (never, ever, or current), AHEI score (quartile), body mass index (continuous), physical activity (<3.0, 3.0-8.9, 9.0-17.9, 18.0-26.9, ≥27.0 MET-hours/week), alcohol consumption (0, 0.1-4.9, 5.0-9.9, 10.0-14.9, ≥15.0 g/day), and regular aspirin use (yes or no). | | | | | | |
| ^b^ SD was the standard deviation of log-transformed hormone levels: 0.58 for estrone, 0.79 for total estradiol, 0.84 for free estradiol, 0.50 for total testosterone, 0.64 for free testosterone, 0.62 for SHBG, and 0.80 for the ratio of total estradiol to total testosterone. | | | | | | |

| Table S5. Associations of plasma sex hormones with conventional adenoma and serrated polyp in postmenopausal women who were selected as controls in the source case-control studies from the NHSI and NHSII | | | | | | | | |
| --- | --- | --- | --- | --- | --- | --- | --- | --- |
| Biomarker | Conventional adenoma | | |  | Serrated polyp | | | *P* for heterogeneity |
|  | N | OR (95% CI) per 1-SD^a^ | *P* |  | N | OR (95% CI) per 1-SD^a^ | *P* |  |
| Estrone |  |  |  |  |  |  |  |  |
| Model 1 | 209 | 1.07(0.93-1.23) | 0.32 |  | 145 | 1.05(0.90-1.23) | 0.54 | 0.65 |
| Model 2 | 209 | 1.12(0.96-1.31) | 0.15 |  | 145 | 1.05(0.87-1.25) | 0.63 | 0.37 |
| Total estradiol |  |  |  |  |  |  |  |  |
| Model 1 | 224 | 1.13(0.96-1.34) | 0.15 |  | 158 | 1.20(0.98-1.46) | 0.08 | 0.82 |
| Model 2 | 224 | 1.23(1.00-1.51) | 0.05 |  | 158 | 1.20(0.94-1.54) | 0.15 | 0.63 |
| Free estradiol |  |  |  |  |  |  |  |  |
| Model 1 | 199 | 1.20(1.00-1.44) | 0.05 |  | 143 | 1.25(1.01-1.55) | 0.04 | 0.86 |
| Model 2 | 199 | 1.28(1.03-1.60) | 0.03 |  | 143 | 1.20(0.93-1.55) | 0.17 | 0.50 |
| Total testosterone |  |  |  |  |  |  |  |  |
| Model 1 | 359 | 0.95(0.86-1.06) | 0.39 |  | 272 | 0.99(0.87-1.12) | 0.84 | 0.59 |
| Model 2 | 359 | 0.94(0.84-1.05) | 0.24 |  | 272 | 0.96(0.84-1.09) | 0.50 | 0.72 |
| Free testosterone |  |  |  |  |  |  |  |  |
| Model 1 | 327 | 1.19(1.05-1.34) | 0.01 |  | 252 | 1.15(1.00-1.33) | 0.04 | 0.57 |
| Model 2 | 327 | 1.12(0.98-1.28) | 0.10 |  | 252 | 0.99(0.85-1.16) | 0.95 | 0.13 |
| SHBG |  |  |  |  |  |  |  |  |
| Model 1 | 361 | 0.73(0.66-0.81) | <.0001 |  | 277 | 0.80(0.71-0.91) | 0.00 | 0.09 |
| Model 2 | 361 | 0.73(0.64-0.82) | <.0001 |  | 277 | 0.90(0.77-1.04) | 0.16 | 0.01 |
| Total estradiol/total testosterone |  |  |  |  |  |  |  |  |
| Model 1 | 206 | 1.11(0.94-1.31) | 0.23 |  | 146 | 1.16(0.95-1.42) | 0.15 | 0.85 |
| Model 2 | 206 | 1.19(0.97-1.46) | 0.09 |  | 146 | 1.19(0.93-1.53) | 0.17 | 0.69 |
| Abbreviations: NHS, the Nurses’ Health Study; OR, odd ratio; CI, confidence interval; SHBG, sex hormone-binding globulin; MET, metabolic equivalent task; AHEI, Alternative Healthy Eating Index.  ^a^ Model 1 was adjusted for age (continuous), fasting status (yes or no), time period of endoscopy (in 2-year intervals), number of prior endoscopies (continuous), and time in years since the most recent endoscopy (continuous); Model 2 was additionally adjusted for race (Caucasian or non-Caucasian), family history of colorectal cancer (yes or no), height (continuous), smoking status (never, ever, or current), AHEI score (quartile), body mass index (continuous), physical activity (<3.0, 3.0-8.9, 9.0-17.9, 18.0-26.9, ≥27.0 MET-hours/week), alcohol consumption (0, 0.1-4.9, 5.0-9.9, 10.0-14.9, ≥15.0 g/day), regular aspirin use (yes or no), and postmenopausal hormone therapy (never, ever, or current). P for heterogeneity was calculated through case-only analysis by comparing serrated polyp with conventional adenoma. SD was the standard deviation of log-transformed hormone levels: 0.58 for estrone, 0.79 for total estradiol, 0.84 for free estradiol, 0.50 for total testosterone, 0.64 for free testosterone, 0.62 for SHBG, and 0.80 for the ratio of total estradiol to total testosterone. | | | | | | | | |

| Table S6. Associations of plasma sex hormones with conventional adenoma and serrated polyp after further adjustment for plasma C-peptide levels in postmenopausal women from the NHSI and NHSII | | | | | | | | |
| --- | --- | --- | --- | --- | --- | --- | --- | --- |
| Biomarker | Conventional adenoma | | |  | Serrated polyp | | | *P* for heterogeneity |
|  | N | OR (95% CI) per 1-SD^a^ | *P* |  | N | OR (95% CI) per 1-SD^a^ | *P* |  |
| Estrone |  |  |  |  |  |  |  |  |
| Model 2+C-peptide | 121 | 1.13(0.91-1.40) | 0.25 |  | 78 | 0.88(0.67-1.15) | 0.36 | 0.50 |
| Total estradiol |  |  |  |  |  |  |  |  |
| Model 2+C-peptide | 131 | 1.07(0.85-1.36) | 0.55 |  | 82 | 0.92(0.68-1.24) | 0.57 | 0.41 |
| Free estradiol |  |  |  |  |  |  |  |  |
| Model 2+C-peptide | 122 | 1.11(0.86-1.42) | 0.42 |  | 78 | 0.99(0.73-1.34) | 0.94 | 0.99 |
| Total testosterone |  |  |  |  |  |  |  |  |
| Model 2+C-peptide | 212 | 0.94(0.81-1.09) | 0.43 |  | 131 | 0.86(0.71-1.03) | 0.11 | 0.20 |
| Free testosterone |  |  |  |  |  |  |  |  |
| Model 2+C-peptide | 201 | 1.10(0.93-1.29) | 0.27 |  | 129 | 1.03(0.83-1.28) | 0.77 | 0.62 |
| SHBG |  |  |  |  |  |  |  |  |
| Model 2+C-peptide | 223 | 0.75(0.64-0.88) | 0.0003 |  | 146 | 0.76(0.62-0.94) | 0.01 | 0.99 |
| Total estradiol/total testosterone |  |  |  |  |  |  |  |  |
| Model 2+C-peptide | 123 | 1.03(0.81-1.30) | 0.82 |  | 78 | 1.02(0.77-1.37) | 0.87 | 0.98 |
| Abbreviations: NHS, the Nurses’ Health Study; OR, odd ratio; CI, confidence interval; SHBG, sex hormone-binding globulin; MET, metabolic equivalent task; AHEI, Alternative Healthy Eating Index.  ^a^ Model 2 was adjusted for age (continuous), fasting status (yes or no), time period of endoscopy (in 2-year intervals), number of prior endoscopies (continuous), time in years since the most recent endoscopy (continuous), race (Caucasian or non-Caucasian), family history of colorectal cancer (yes or no), height (continuous), smoking status (never, ever, or current), AHEI score (quartile), body mass index (continuous), physical activity (<3.0, 3.0-8.9, 9.0-17.9, 18.0-26.9, ≥27.0 MET-hours/week), alcohol consumption (0, 0.1-4.9, 5.0-9.9, 10.0-14.9, ≥15.0 g/day), and regular aspirin use (yes or no). P for heterogeneity was calculated through case-only analysis by comparing serrated polyp with conventional adenoma. SD was the standard deviation of log-transformed hormone levels: 0.58 for estrone, 0.79 for total estradiol, 0.84 for free estradiol, 0.50 for total testosterone, 0.64 for free testosterone, 0.62 for SHBG, and 0.80 for the ratio of total estradiol to total testosterone. | | | | | | | | |

| Table S7. Associations of plasma sex hormones with conventional adenoma and serrated polyp in postmenopausal women according to the median time interval since blood draw | | | | | | | | |
| --- | --- | --- | --- | --- | --- | --- | --- | --- |
| Biomarker | Conventional adenoma | | |  | Serrated polyp | | | *P* for heterogeneity |
|  | N | OR (95% CI) per 1-SD^a^ | *P* |  | N | OR (95% CI) per 1-SD^a^ | *P* |  |
| <11 years |  |  |  |  |  |  |  |  |
| Estrone | 142 | 1.16(0.96-1.40) | 0.13 |  | 96 | 0.83(0.68-1.03) | 0.09 | 0.01 |
| Total estradiol | 152 | 1.19(0.93-1.52) | 0.17 |  | 107 | 0.96(0.72-1.29) | 0.78 | 0.18 |
| Free estradiol | 134 | 1.24(0.95-1.61) | 0.12 |  | 96 | 1.12(0.83-1.52) | 0.44 | 0.69 |
| Total testosterone | 239 | 0.94(0.83-1.07) | 0.37 |  | 189 | 0.89(0.76-1.03) | 0.11 | 0.64 |
| Free testosterone | 223 | 1.12(0.96-1.31) | 0.15 |  | 178 | 1.07(0.89-1.28) | 0.48 | 0.62 |
| SHBG | 246 | 0.80(0.70-0.92) | 0.002 |  | 195 | 0.80(0.68-0.94) | 0.01 | 0.86 |
| Total estradiol/total testosterone | 139 | 1.01(0.79-1.29) | 0.93 |  | 98 | 1.07(0.80-1.44) | 0.63 | 0.64 |
| ≥11 years |  |  |  |  |  |  |  |  |
| Estrone | 143 | 1.04(0.86-1.25) | 0.70 |  | 97 | 1.23(0.98-1.56) | 0.08 | 0.23 |
| Total estradiol | 154 | 1.05(0.86-1.27) | 0.64 |  | 105 | 1.18(0.95-1.48) | 0.14 | 0.45 |
| Free estradiol | 143 | 1.15(0.94-1.41) | 0.19 |  | 98 | 1.10(0.86-1.40) | 0.44 | 0.66 |
| Total testosterone | 247 | 0.94(0.82-1.07) | 0.33 |  | 180 | 1.00(0.85-1.17) | 0.97 | 0.57 |
| Free testosterone | 226 | 1.12(0.96-1.31) | 0.15 |  | 168 | 1.01(0.85-1.21) | 0.88 | 0.27 |
| SHBG | 248 | 0.74(0.64-0.85) | <.0001 |  | 183 | 0.93(0.77-1.13) | 0.48 | 0.02 |
| Total estradiol/total testosterone | 146 | 1.07(0.89-1.30) | 0.47 |  | 100 | 1.06(0.85-1.33) | 0.59 | 0.62 |
| Abbreviations: OR, odd ratio; CI, confidence interval; SHBG, sex hormone-binding globulin; MET, metabolic equivalent task; AHEI, Alternative Healthy Eating Index.  ^a^ Adjusted for age (continuous), fasting status (yes or no), time period of endoscopy (in 2-year intervals), number of prior endoscopies (continuous), time in years since the most recent endoscopy (continuous), race (Caucasian or non-Caucasian), family history of colorectal cancer (yes or no), height (continuous), smoking status (never, ever, or current), AHEI score (quartile), body mass index (continuous), physical activity (<3.0, 3.0-8.9, 9.0-17.9, 18.0-26.9, ≥27.0 MET-hours/week), alcohol consumption (0, 0.1-4.9, 5.0-9.9, 10.0-14.9, ≥15.0 g/day), regular aspirin use (yes or no), and postmenopausal hormone therapy (never, ever, or current). P for heterogeneity was calculated through case-only analysis by comparing serrated polyp with conventional adenoma. SD was the standard deviation of log-transformed hormone levels: 0.58 for estrone, 0.79 for total estradiol, 0.84 for free estradiol, 0.50 for total testosterone, 0.64 for free testosterone, 0.62 for SHBG, and 0.80 for the ratio of total estradiol to total testosterone. | | | | | | | | |
